# Appleton’s Journal of Literature, Science and Art

**Published:** 1873-03

**Authors:** 


*Appleton's Journal of Literature, Science and Art*

Is a weekly journal, abounding with fine illustrations and overflowing with literary "good things." All readers will prize *Appleton's* for its high tone, pure literature and elevated sentiments. It will warm the home-circle with its bright and genial weekly visits. D. Appleton & Co., 549 and 551 Broadway, New York. Price, $4 per annum.

